# Abscisic Acid Regulates Auxin Homeostasis in Rice Root Tips to Promote Root Hair Elongation

**DOI:** 10.3389/fpls.2017.01121

**Published:** 2017-06-28

**Authors:** Tao Wang, Chengxiang Li, Zhihua Wu, Yancui Jia, Hong Wang, Shiyong Sun, Chuanzao Mao, Xuelu Wang

**Affiliations:** ^1^National Key Laboratory of Genetic Engineering, School of Life Sciences, Fudan UniversityShanghai, China; ^2^National Key Laboratory of Crop Genetic Improvement, Center of Integrative Biology, College of Life Science and Technology, Huazhong Agricultural UniversityWuhan, China; ^3^State Key Laboratory of Plant Physiology and Biochemistry, College of Life Science, Zhejiang UniversityHangzhou, China

**Keywords:** ABA, auxin, crosstalk, root hair elongation, transport, biosynthesis, *Oryza sativa*

## Abstract

Abscisic acid (ABA) plays an essential role in root hair elongation in plants, but the regulatory mechanism remains to be elucidated. In this study, we found that exogenous ABA can promote rice root hair elongation. Transgenic rice overexpressing *SAPK10* (*Stress/ABA-activated protein kinase 10*) had longer root hairs; rice plants overexpressing *OsABIL2* (*OsABI-Like 2*) had attenuated ABA signaling and shorter root hairs, suggesting that the effect of ABA on root hair elongation depends on the conserved PYR/PP2C/SnRK2 ABA signaling module. Treatment of the *DR5-GUS* and *OsPIN-GUS* lines with ABA and an auxin efflux inhibitor showed that ABA-induced root hair elongation depends on polar auxin transport. To examine the transcriptional response to ABA, we divided rice root tips into three regions: short root hair, long root hair and root tip zones; and conducted RNA-seq analysis with or without ABA treatment. Examination of genes involved in auxin transport, biosynthesis and metabolism indicated that ABA promotes auxin biosynthesis and polar auxin transport in the root tip, which may lead to auxin accumulation in the long root hair zone. Our findings shed light on how ABA regulates root hair elongation through crosstalk with auxin biosynthesis and transport to orchestrate plant development.

## Introduction

Roots have important functions in uptake of nutrients and water, and anchoring plants in the soil. Root hairs, extensions from single root epidermal cells, constitute up to an estimated 70% of the root surface area in crops (Richardson et al., 2009; Pereg and McMillan, 2015). Root hairs help plants maintain sufficient levels of water and nutrients; for example, in different plant species under phosphate (P)-limiting conditions, up to 90% of the mineral nutrients appear to be taken up by root hairs (Föhse et al., 1991). In addition, root hairs play important roles in the uptake and transport of NO_3_^-^ and NH_4_^+^ (Gilroy and Jones, 2000). Compared to bald roots, a root 1 mm in diameter with root hairs 0.5 or 1 mm in average length, growing in sand, will improve the soil water uptake rate by 30 to 55% in barley (Segal et al., 2008).

Phytohormones and abiotic stresses affect root hair formation and elongation in *Arabidopsis* and crops. ABA, a major abiotic stress-responsive hormone, plays an important role in root hair elongation. The application of exogenous ABA leads to root swelling and root hair formation in the tips of young and seminal rice roots, and this process requires *de novo* synthesis of proteins (Chen et al., 2006). Rice and *Arabidopsis* plants under moderate water stress (treated with polyethylene glycol) accumulate ABA and grow root hairs with high intensity (Xu et al., 2013).

Work in *Arabidopsis* has identified a core ABA signaling pathway (Ma et al., 2009; Park et al., 2009; Umezawa et al., 2009; Cutler et al., 2010). The ABA receptors PYRABACTIN RESISTANCE1 (PYR1)/PYRABACTIN RESISTANCE1-LIKE (PYL)/REGULATORY COMPONENTS OF ABA RECEPTOR (RCAR) bind to ABA and then interact with the subclass A type 2C protein phosphatases (PP2Cs), which can suppress SNF1-RELATED PROTEIN KINASE 2 (SnRK2s). As a result, SnRK2s phosphorylate and activate bZIP transcription factors to regulate ABA-responsive gene expression.

Bioinformatics work in rice has identified orthologs of these *Arabidopsis* ABA signaling components (Kim et al., 2012; He et al., 2014). Rice contains 10 members of the OsPYL/RCAR family, 9 members of the subclass A PP2Cs, 10 SAPKs (Stress/ABA-activated protein kinases), and 10 members of the group A bZIP transcription factors (Kim et al., 2012). *OsABIL2*, a rice ortholog of *AtABI1* and *AtABI2*, plays a negative role in rice ABA signaling, as OsABIL2 can interact with and dephosphorylate SAPK10 to form an OsPYL1–OsABIL2–SAPK8/10 ABA signaling module (Li et al., 2015). Furthermore, the root hair length of *OsABIL2* overexpression lines is significantly reduced (Li et al., 2015). However, little is known about the underlying cellular and molecular mechanisms of ABA in regulating root hair development.

Auxin also regulates root hair elongation. Exogenous auxin enhances root hair length, and inhibition of auxin signaling represses root hair elongation (Pitts et al., 1998; Rahman et al., 2002). In *Arabidopsis*, active polar auxin transport moves auxin to the root tip to regulate root hair elongation, and blocking auxin transport results in short root hair (Pitts et al., 1998; Rahman et al., 2002). Auxin efflux mediated by PIN2 facilitates auxin supply through basipetal auxin transport from the root apex to the root hair differentiation zone (Cho et al., 2007; Cheng et al., 2013). Modeling of auxin flow suggests that auxin influx carrier AUX1-dependent transport through non-hair cells can maintain auxin supply for developing hair cells and sustain root hair outgrowth (Jones et al., 2009). These studies showed that changes in the endogenous or exogenous auxin content through auxin transport in root tip and root hair cells affected root hair elongation.

Formation of the auxin gradient in root tips depends on auxin transport and requires local auxin biosynthesis. Auxin can be synthesized locally in the root tip (Ljung et al., 2005; Petersson et al., 2009), and a simple two-step pathway that converts tryptophan to IAA acts as the main auxin biosynthesis pathway in *Arabidopsis* (Mashiguchi et al., 2011; Zhao, 2012). Trp is first converted to indole-3-pyruvate (IPA) by the TAA family of amino transferases, and IPA is converted into IAA by the YUCCA (YUC) family of flavin monooxygenases. Accordingly, overexpression of the auxin biosynthesis gene *YUCCA1* in *Arabidopsis* enhanced root hair growth compared to wild type (Zhao et al., 2001) and inhibition of auxin biosynthesis using L-amino-oxyphenypropionic acid (AOPP) blocked the formation of root hairs, which can be rescued by the application of exogenous IAA (Soeno et al., 2010).

Many studies have suggested that ABA interacts with auxin to regulate root growth and development (Kobayashi et al., 2005; Vanstraelen and Benková, 2012). For example, the mutants of *AUXIN RESPONSE FACTOR 2* (*ARF2*), which affects auxin-mediated responses, show enhanced ABA sensitivity during seed germination and primary root growth, and ABA treatment alters auxin distribution in *Arabidopsis* primary root tips (Wang et al., 2011). *ABI4* mediates ABA’s inhibition of lateral root formation via reduction of polar auxin transport, resulting in decreased auxin levels in roots (Shkolnik-Inbar and Bar-Zvi, 2010). Auxin may act as an organizer of hormonal signals for root hair growth (Lee and Cho, 2013). However, it remains unclear whether the ABA-induced root hair elongation in rice occurs through polar auxin transport or local auxin biosynthesis in the root tip.

In this study, we used transgenic lines with enhanced ABA signaling (*SAPK10* overexpression) or attenuated ABA signaling (*OsABIL2* overexpression) to study how ABA signaling regulates root hair elongation. We found that ABA signaling promotes root hair length in root tips and that the ABA-promoted root hair elongation requires polar auxin transport. Our RNA-seq analysis found that ABA enhances both auxin transport and auxin biosynthesis in root tips and identified a set of genes co-regulated by ABA and auxin to promote root hair length.

## Materials and Methods

### Plant Materials and Growth Conditions

The wild-type rice Dongjin (*O. sativa* L. cv. *japonica*) was used in this work. The *OsABIL2* and *OsSAPK10* overexpression transgenic lines were in the Dongjin background, and the detail of *OsABIL2* overexpression transgenic lines were introduced in previous study (Li et al., 2015). The auxin-related transgenic rice lines *DR5-GUS* (ZH11 background), *OsPIN1b-GUS, OsPIN1c-GUS, OsPIN2-GUS, OsPIN5a-GUS*, and *OsPIN10a-GUS* were from a previous study (Wang et al., 2009). For polar auxin transport assays by crown root GUS staining, the F_1_ hybrid of *OsABIL2-OE* X *DR-GUS* and DJ X *DR5-GUS* lines was used.

For physiological analysis, the rice seeds were imbibed for 2 days at 30°C, and the germinated seeds were then transferred to bottom-cut 96-well PCR plates. Seedlings were grown on water with a 16-h (light, 28°C)/8-h (dark, 25°C) rhythm for the indicated days, which was defined as normal conditions in this study.

### Generation of Transgenic Rice Plants

For overexpressing *SAPK10*, the genomic sequence of *Os03g0610900* was cloned into the binary vector *pCAMBIA1306* fused with a FLAG-tag at the C-terminus. The primers used for cloning *OsSAPK10* are shown in Supplementary Table 1.

### Phytohormone and Chemical Treatments of Plant Materials

For measurement of *SAPK10-OE* expression level, roots of the 7-day-old wild-type and transgenic seedlings grown under normal conditions were cut, and the samples were frozen in liquid nitrogen and stored at -80°C for RNA extraction.

For root hair induction assays, the 5-day-old normal grown seedlings were transferred to solutions with or without the following additions: ABA (100 mM dissolved in ethanol and in order to add the same amount of ethanol between treatment group and mock, we gradient dilution ABA to 20 mM, 10 mM, 5 mM, 2 mM, and 1 mM solution), NAA (100 mM dissolved in ethanol, and in order to add the same amount of ethanol, we gradient dilution NAA to 5 and 1 mM solution), the ABA biosynthesis inhibitor fluridon (FLU) (100 mM dissolved in ethanol), the auxin efflux inhibitor NPA (100 mM dissolved in DMSO), and the control added the same amount of ethanol and/or DMSO. After 24 h, the crown roots were fixed in FAA solution (ethanol: acetic acid: 37% formaldehyde: H_2_O = 50:5:10:35, v/v), and then photographed with a stereoscopic microscope (Carl Zeiss Discovery V20).

For β-glucuronidase (GUS) staining, crown roots were immersed in the GUS staining solution (1 mg/ml X-glucuronide in 100 mM sodium phosphate, pH 7.2, 0.5 mM ferricyanide, 0.5 mM ferrocyanide, and 0.1% Triton X-100), briefly subjected to a vacuum, and then incubated at 37°C in the dark. The stained plant roots were photographed using Carl Zeiss Discovery V20 stereomicroscope.

### Scanning Electron Microscopy (SEM)

Crown roots of the 6-day-old seedlings were cut and fixed in FAA solution overnight. The fixed samples were dehydrated in an ethanol series of 50, 60, 70, 80, 90, and 100% ethanol for 1 h each, and then treated with ethanol: tert-butyl alcohol (v/v), 3:1, 1:1, and 1:3 for 1 h each. Finally, the samples were kept in pure tert-butyl alcohol. After vacuum freeze-drying, the samples were sputter-coated with Au-Pd and further visualized with a scanning electron microscope (Hitachi TM3000). For observation of root hair initiation, we screened the root tip from the root apex to the root hair zone.

### Root-Hair Length Measurement

Root hairs were observed, and images were captured with a Discovery V20 Stereomicroscope (Carl Zeiss). The zone of root hair growth is 4–5 mm from the root apex. At least 15 crown roots were measured, and 20 root hairs of each root were measured. Root-hair length measurements were performed with ImageJ^1^.

### Quantification of IAA

To analyze IAA concentrations, firstly, according to the root hair phenotype, we divided the root tips of rice crown roots into three regions: short root hair zone (SRH), long root hair zone (LRH), and root tip zone (Tip) (Supplementary Figure 3). Each region was excised from at least 96 five-day-old seedlings treated with 0.5 μM ABA for 24 h, or not treated as a control. Samples were separated under Carl Zeiss Discovery V20 stereomicroscope and immediately frozen in liquid nitrogen, afterward stored at -80°C. Samples were powdered in liquid nitrogen and at least 30 mg powder homogenized in 750 μl of cold extraction buffer 1 [methanol: H2O: acetonitrile = 80:19:1 (v/v), 10 ng/ml d5-IAA], then shade and shake at 4°C 16 h (300 rpm). Samples were centrifuged at 4°C at 13,000 rpm for 10 min, the supernatant is transferred to another Eppendorf tube. Add 450 μl of cold extraction buffer 2 [methanol: H2O: acetonitrile = 80:19:1 (v/v)] into precipitate, shade and shake at 4°C 4 h (300 rpm), centrifuged at 4°C at 13,000 rpm for 10 min, combine two supernatants, transfer supernatant through 0.22 μm filter to a new Eppendorf tube. Then each sample was dried using nitrogen flow and re-dissolved in 200 μl of 30% cold methanol 3–6 h. Quantification was performed in an ABI 4000Q-TRAR LC-MS system (Applied Biosystems, United States) with stable, isotope-labeled auxin as the standard (OlChemIm, Czech Specials) according to a method described previously (Liu et al., 2012).

### Quantitative Real-Time PCR Assays

The qRT-PCR assays were carried out as described previously with small modifications (Zhang et al., 2009). The primers for qRT-PCR were designed with NCBI Primer-Blast^2^ to avoid the homologous regions, shown in Supplementary Table 1. Total RNA was extracted with the Plant RNAprep Kit (Tiangen). About 2 μg DNase-treated RNA was used for reverse transcription (M-MLV reverse transcriptase, TaKaRa). The RT-PCR amplifications were carried out with a Bio-Rad CFX system, and PCR products were monitored with SYBR green dye. The expression level was normalized by the expression of *OsACTIN1* (*LOC_Os03g50885*, internal control), and RT-qPCR results were analyzed by the 2^-ΔΔCT^ method using Bio-Rad CFX Manager 3.1 software.

### Sample Collection and RNA Isolation for RNA-Seq

According to the root hair phenotype, we divided the root tips of rice crown roots into three regions: short root hair zone (SRH), long root hair zone (LRH), and root tip zone (Tip). Each region was excised from at least 96 five-day-old seedlings treated with 0.5 μM ABA for 24 h, or not treated as a control. Samples were separated and immediately frozen in liquid nitrogen, and afterward stored at -80°C until RNA isolation. Total RNA was extracted from each tissue using TRIzol (Invitrogen). RNA purity was checked with a NanoPhotometer spectrophotometer (IMPLEN, Munich, Germany) and RNA integrity was assessed with an Agilent 2100 Bioanalyzer (Agilent, Palo Alto, CA, United States).

### The cDNA Library Preparation and Transcriptome Sequencing

Two biological replicates were used for RNA-seq experiments for each tissue type. A total amount of 3 μg RNA per sample was used as input material for the RNA sample preparations. The samples were sent to Beijing Novogene Bioinformatics Technology Co., Ltd. (Beijing, China). The sequencing libraries were constructed using a NEBNext Ultra RNA Library Prep Kit for Illumina (NEB, Ipswich, MA, United States) following the manufacturer’s recommendations, and index codes were added to attribute sequences to each sample. Briefly, mRNA was purified from total RNA using poly(T) magnetic beads. Quality of these libraries was assessed with an Agilent 2100 Bioanalyzer system. The clustering of the index-coded samples was performed with a cBot Cluster Generation System using a TruSeq PE Cluster Kit v3-cBot-HS (Illumina, San Diego, CA, United States) according to the manufacturer’s instructions. After cluster generation, the libraries were sequenced on an Illumina Hiseq 4000 platform and 150-bp paired-end reads were generated.

In this study, the genes with a significant (*P* ≤ 0.05) fold change > 2 or < -2 between the control and ABA treatment were defined as differentially expressed genes responding to ABA stimulation.

### Statistical Analysis

Statistical analysis was performed using IBM SPSS 20.0 software. One-way analysis of variance was used, and comparison between the two groups was performed using the least significant difference (LSD) test. Differences were considered significant if *P* < 0.05.

### Functional Enrichment Analysis

Gene ontology analysis was carried out using the Singular Enrichment Analysis (SEA) tool offered by agriGO (Du et al., 2010) at default settings of Fisher *t*-test (*P* < 0.05), False Discovery Rate (FDR) correction by Hochberg method and five minimum number of mapping entries against the rice-specific precomputed background reference.

## Results

### ABA Regulates Root Hair Growth in Rice

**FIGURE 1:**
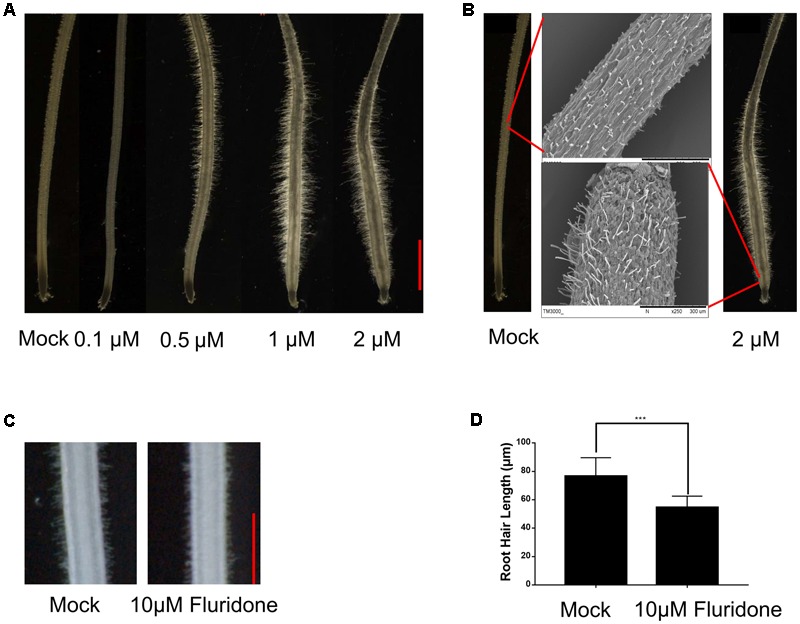
ABA promotes root hair elongation in the rice root tips. **(A)** The 5-day-old rice root tips grown in solution containing the indicated concentrations of ABA for 24 h. Scale bar = 1 mm. **(B)** Morphology of root hair initiation zones in the 6-day-old crown roots treated with mock or 2 μM ABA for 24 h. **(C)** Root hair morphology of the wild type with or without Fluridon (10 μM) treatment. Scale bar = 500 μm. **(D)** Quantification of root hair length of the wild type with or without Fluridon treatment. Data are means ± SD.

To understand how plants adapt to environmental stresses, we tested whether the stress-responsive hormone ABA affects root hair growth in the Dongjin (DJ) cultivar of rice. Crown roots of the 5-day-old seedling were treated with exogenous ABA at concentrations of 0.1, 0.5, 1, and 2 μM for 24 h. As shown in **Figure 1A**, in the seedlings treated with 0.1 μM ABA, the root hair length was significantly less than in the control seedlings not treated with ABA. However, treatments with 0.5, 1, and 2 μM ABA significantly enhanced root hair elongation, indicating higher concentrations of ABA can promote root hair elongation, and we used the ABA concentrations of 0.5 and 2 μM for further studies.

We also used SEM to observe root hair morphology. We found that most root hairs initiated in the region about 3 mm from the root apex under normal growth conditions, but most root hairs initiated in the region about 0.2 mm from the apex in roots treated with 2 μM ABA for 24 h. We also further confirmed that the root hair length increased in response to ABA treatment (**Figure 1B** and Supplementary Figure 1). These results indicated that exogenous ABA not only enhances root hair length, but also promotes earlier root hair initiation. In addition, fluridone as an ABA biosynthetic inhibitor (10 μM) was used to investigate the effect of ABA on root tip responses under moderate water stress in rice and *Arabidopsis* (Yoshioka et al., 1998; Xu et al., 2013). The treatment of fluridon significantly repress the root hair length (**Figures 1C,D**). We measured the root hair length which located 4–5 mm distant from root apex in **Figure 1D**, this because considering the several ABA concentration treatment of root hair phenotype, we found that the best region to measure root hair length located at 4–5 mm distant from root tip.

### ABA Signaling Promotes Root Hair Elongation

**FIGURE 2:**
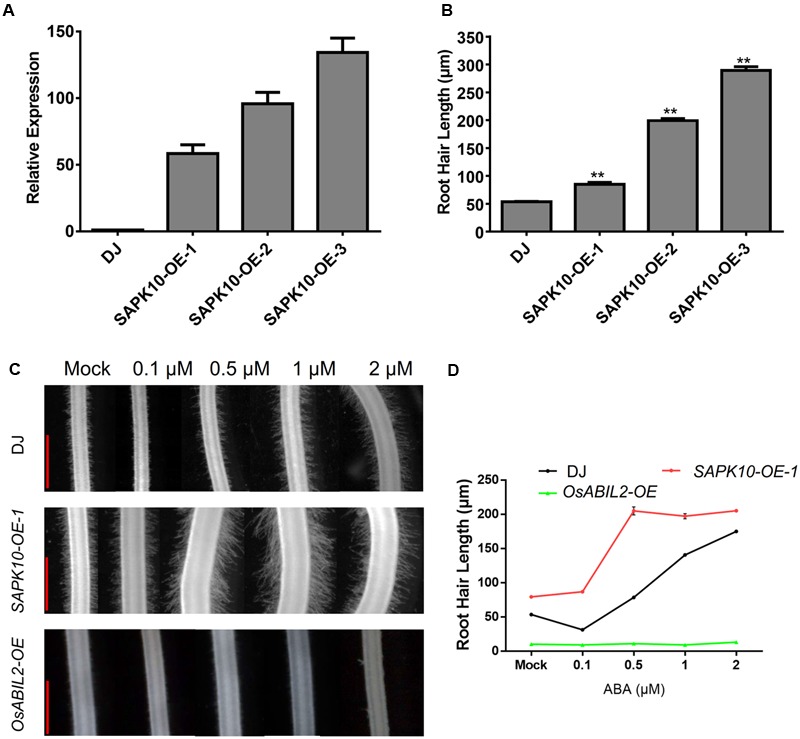
ABA-induced root hair elongation is dependent on ABA signaling. **(A)** Expression level of *SAPK10* in the *SAPK10-OE* lines determined by quantitative RT-PCR. Seven-day-old roots were used. **(B)** Root hair length of the *SAPK10-OE* line. The root hairs located about 4–5 mm from the root apex were measured. About 20 longest root hairs from each of the 15 crown roots were measured. Data are means ± SE. **(C)** Root hair morphology of the wild type, and the *SAPK10-OE-1* and *OsABIL2-OE* transgenic lines. Five-day-old seedlings were treated with different concentrations (0, 0.1, 0.5, 1, and 2 μM) of ABA for 24 h. Scale bar = 500 μm. **(D)** Quantification of root hair length of the wild type, *SAPK10-OE*, and *OsABIL2-OE* lines. Data are means ± standard error (SE).

To investigate whether ABA promotes root hair elongation through the major ABA signaling components, we constructed rice lines overexpressing *SAPK10*, which plays a positive role in the ABA signaling. Three of the *SAPK10* overexpression lines showed ∼60-fold, ∼100-fold, and ∼130-fold increases in *SAPK10* expression in the roots of the 7-day-old seedlings (**Figure 2A**). Then we measured the length of root hairs in these lines and found that the *SAPK10* overexpression significantly enhanced root hair elongation (**Figure 2B** and Supplementary Figure 2). However, root hairs were rarely observed in the transgenic lines expressing *OsABIL2*, which is a negative regulator of rice ABA signaling (**Figure 2C**). The next to detect the root hair of *SAPK10-OE* and *OsABIL2-OE* response to ABA treatment, we would like to clarify the *SAPK10-OE-1* was used in this study. Because this *SAPK10*-*OE-1* line has moderate overexpression level of *SAPK10* gene, which lead to moderate response to ABA treatment suitable for comparison with the wild type. While the other two lines *SAPK10-OE-2*, and *-3* have much higher level expression of *SAPK10* than the *SAPK10-OE-1* line (**Figure 2A**), which lead to root hair elongation in the region very close to the root tip in the other two lines, rather than in the region 4–5 mm from the root tip after ABA treatment (Supplementary Figure 2). Considering these factors, we choose the *SAPK10-OE-1* line for all other experiments for better comparison to the wild type after ABA treatments. With the 0, 0.1, 0.5, 1, and 2 μM ABA treatments (**Figures 2C,D**), the root hair length of the *SAPK10-OE-1* line increased much more than that of the wild-type DJ. In contrast, ABA treatment did not increase root hair elongation of the *OsABIL2-OE* plants, which is a negative regulator of rice ABA signaling. This indicated that the effect of ABA on root hair elongation largely depends on the major PYR/PP2C/SnRK2 signaling pathway.

### Polar Auxin Transport Is Required for the ABA-Mediated Root Hair Elongation

**FIGURE 3:**
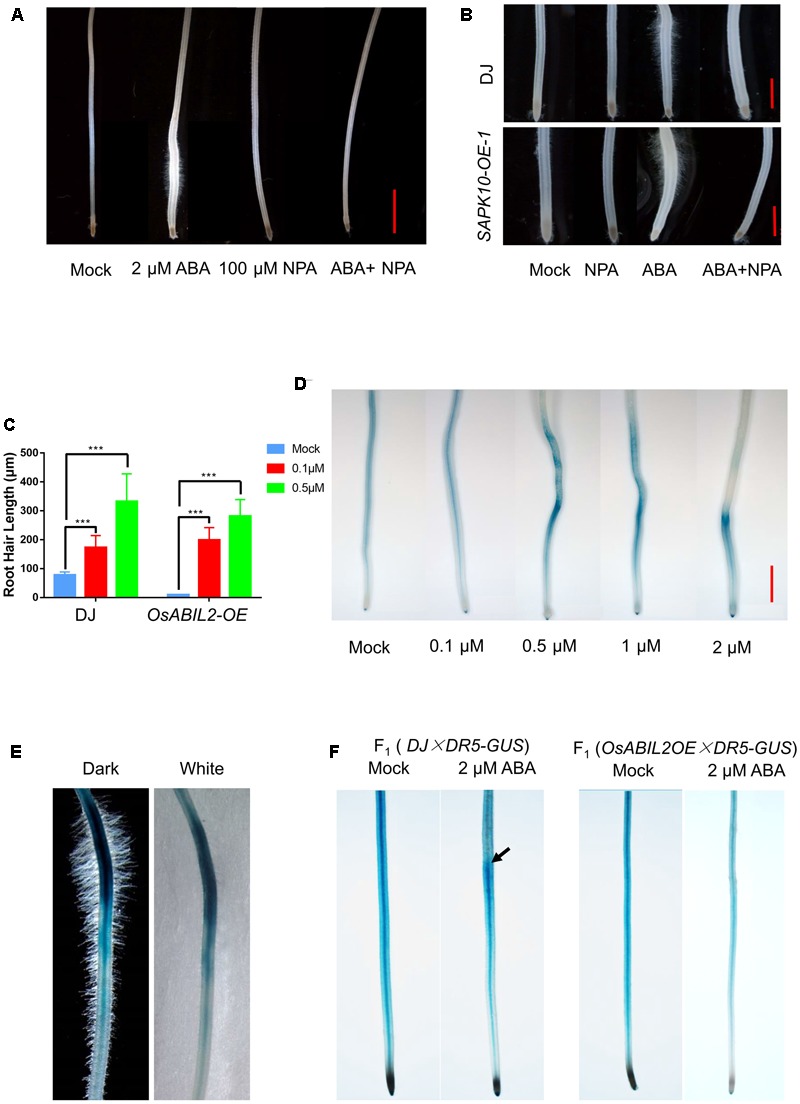
Auxin acts downstream of ABA signaling to promote root hair elongation. **(A)** Effect of the polar auxin transport inhibitor NPA on root hair elongation. Five-day-old seedlings were grown in solutions containing the indicated concentrations of ABA and/or NPA for 24 h. Scale bar = 2 mm. **(B)** Root hair elongation of the *SAPK10-OE* plants in response to ABA and NPA treatments. Five-day-old seedlings were grown in solutions containing the indicated concentrations of ABA and/or NPA for 24 h. Scale bar = 1 mm. **(C)** Quantification of root hair length of the *OsABIL2-OE* plants and wild type in response to the exogenous auxin. Five-day-old seedlings were grown in solutions containing 0, 0.1, or 0.5 μM NAA for 24 h. Data are means ± SD. **(D)** ABA affects the concentration and distribution of auxin indicated by the *DR5-GUS* reporter. Five-day-old seedlings were grown in solutions containing different concentrations of ABA for 24 h. Scale bar = 2 mm. **(E)** Close-up view of the long root hair zone of the *DR5-GUS* line treated with 0.5 μM ABA for 24 h. Images with dark (left) and white (right) backgrounds are shown. **(F)** The GUS activity in the F_1_ seedlings in response to treatment with 2 μM ABA for 24 h. Wild type (DJ) and the *OsABIL2-OE* plants were crossed with the *DR5-GUS* line (Zhonghua11 background, 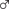). Five-day-old seedlings were used. Black arrows indicate the accumulation of DR5-GUS in the long root hair zone.

Auxin has a positive effect on root hair elongation without affecting the determination of root hair cell fate in *Arabidopsis* (Masucci and Schiefelbein, 1994, 1996; Pitts et al., 1998; Cho and Cosgrove, 2002). Therefore, we asked whether the ABA-regulated root hair elongation in rice also requires auxin transport. Then we used the auxin efflux inhibitor NPA to examine this. The 5-day-old wild-type seedlings were treated with ABA, NPA, or ABA plus NPA for 24 h. As shown in **Figure 3A**, ABA plus NPA treatment inhibited root hair elongation as compared with ABA alone. Similarly, in the *SAPK10-OE* line, ABA plus NPA also severely inhibited the root hair elongation as compared with the wild type shown in **Figure 3B**. We then examined whether additional auxin can rescue the reduced root hair elongation in the *OsABIL2-OE*, using the 5-day-old seedlings of *OsABIL2-OE* treated with 0.1 μM or 0.5 μM NAA for 24 h. As shown in **Figure 3C**, compared with Mock, 0.5 μM of NAA can significantly rescue the root hair length of *OsABIL2-OE*. Taken together, these results indicated that auxin acts downstream of ABA to mediate the ABA-induced root hair elongation.

To address whether ABA influences local auxin concentrations, we used a *DR5-GUS* rice line, which has been widely used as a marker line for monitoring endogenous auxin levels in plants (Yamamoto et al., 2007). The 5-day-old *DR5-GUS* seedlings were treated with 0, 0.1, 0.5, 1, or 2 μM ABA for 24 h, then we stained their roots for GUS activity. We found that *DR5* was widely expressed in the crown roots, especially in their steles, and with increased concentrations of exogenous ABA, the GUS staining gradually accumulated close to the root tip and in the outer layers of the roots (**Figure 3D**). With a higher magnification, we observed that the region with the strongest *GUS* signal produced the longest root hairs (**Figure 3E**), suggesting that the ABA-promoted local auxin accumulation may lead to the specified root hair elongation.

We further tested whether the ABA-induced auxin response is dependent on ABA signaling by crossing the *DR5-GUS* into the *OsABIL2-OE* background; the wild type crossed with *DR5*-*GUS* (ZH11 background) was used as a control. The GUS staining showed that ABA treatment did not cause auxin redistribution in the *OsABIL2-OE* crown roots (**Figure 3F**), indicating that ABA signaling promotes auxin accumulation in the root tip to induce root hair elongation. The *DR5-GUS* staining patterns look different between **Figures 3D,F**. There may be two reasons. First, in **Figure 3F**, F_1_ seeds were used for the observation, which contains half dosage of the reporter gene GUS. Therefore, the GUS staining apparently is weaker in **Figure 3F** than in **Figure 3D**. In general, the relative staining density and the trends of *DR5-GUS* expression pattern between **Figures 3D,F** are similar.

### ABA Modulates the Expression Level and Pattern of Genes Involved in Auxin Transport

**FIGURE 4:**
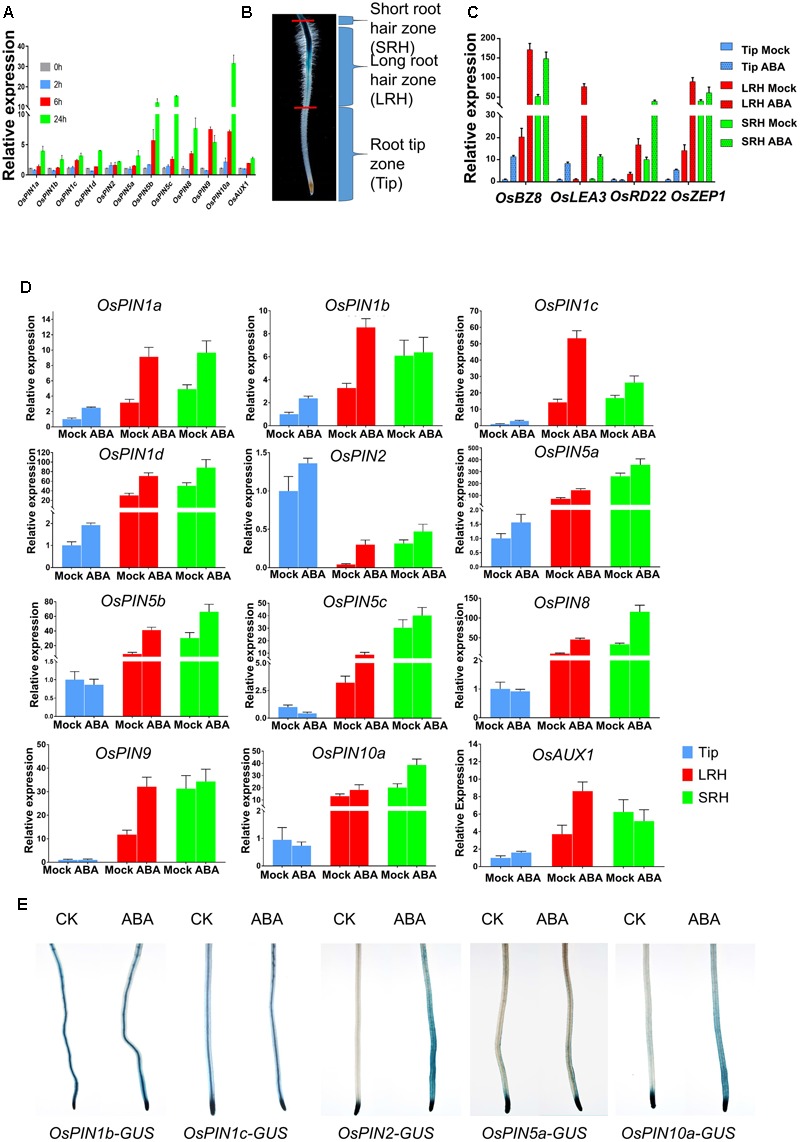
ABA regulates the expression of *OsPINs* to promote polar auxin transport in root tips. **(A)** Expression of *OsPINs* is induced by ABA in 5-day-old rice roots. Expression of each *PIN* gene was determined by quantitative RT-PCR and 2 μM ABA was used in this assay. *OsActin1* was used as an internal control. Three replicates were conducted. Data are means ± SD. **(B)** Definition of the root hair zones. Root tips were divided into three regions: short root hair zone (SRH), long root hair zone (LRH), and root tip zone (Tip). Each region was cut and collected from at least 96 five-day-old seedlings with or without 0.5 μM ABA treatment for 24 h. **(C)** Expression of ABA-responsive genes *OsLEA3, OsBZ8, OsZEP1*, and *OsRD22* in the Tip, LRH and SRH zones after ABA treatment. Data are means ± SD. **(D)** Quantitative RT-PCR analysis of *OsPINs* in the SRH, LRH and Tip treated with or without 0.5 μM ABA for 24 h. The expression level of each gene in the untreated Tips was defined as “1.” Data are means ± SD. **(E)** The GUS activity of the *OsPINs-GUS* in the 5-day-old rice seedlings in response to 0.5 μM ABA for 12 h.

Auxin redistribution is mainly controlled by auxin transporters, which include the influx transporters of the AUXIN1/LIKE AUX1 (AUX1/LAX) family (Kerr and Bennett, 2007; Swarup et al., 2008; Zhao et al., 2015), and efflux transporters of the PIN-FORMED (PIN) families (Petrasek et al., 2006; Wang et al., 2009). To test how ABA signaling regulates auxin transport, we investigated the ABA-regulated expression of the major genes involved in auxin transport. We found that the mRNA levels of *OsPINs* and *OsAUX1* were induced by treatment with 2 μM ABA at 6 and 24 h (**Figure 4A**).

To examine the spatial expression of these genes, we divided the root tip into three zones according to the root hair distribution and DR5-GUS activity: the short root hair zone (SRH), long root hair zone (LRH), and root tip zone (Tip) (**Figure 4B** and Supplementary Figure 3). First, we tested expression of the ABA marker genes *OsLEA3, OsBZ8, OsZEP1*, and *OsRD22* after ABA treatments (**Figure 4C**), and the result showed that all of the ABA marker genes (except *OsRD22* in Tip) were induced in the three different zones. We then detected the expression of *OsPINs* in the three zones and found that most of the *OsPINs* were induced by the ABA treatment (**Figure 4D**). Second, we used the *GUS* reporter lines driven by various *OsPIN* promoters (*OsPIN1b-GUS, OsPIN1c-GUS, OsPIN2-GUS, OsPIN5a-GUS*, and *OsPIN10a-GUS*) (Wang et al., 2009) to visualize the *OsPIN* expression pattern. We found that the expression of *OsPIN1b-GUS, OsPIN1c-GUS*, and *OsPIN5a-GUS* was not dramatically altered by ABA treatment, but the expression of *OsPIN2-GUS* and *OsPIN10a-GUS* was significantly enhanced by ABA treatment, especially in the outer layers of the root (**Figure 4E**). In this assay, we indeed found that there were some different between real-time RT-PCR and GUS staining. In general, real-time RT-PCR or promoter-GUS has their own advantages and disadvantages. First, real-time RT-PCR cannot distinguish the specific expression tissue, which may mask some tissue specific signals. Second, the promoter-GUS approach has a defect on the specificity of promoter, because some times, ones do not known what the exact promoter size in plants. Therefore, the combination of these two approaches will show the expression pattern more accurately. This assay we wanted to show the genes expression fold change used the real-time RT-PCR, and GUS staining to show tissue specificity. In *Arabidopsis, PIN2* is expressed in the epidermal cells (Wisniewska et al., 2006). We also checked the rice microarray expression database (RiceXPro^3^, *OsPIN2* Locus ID: *Os06g0660200, OsPIN10a* Locus ID: *Os01g0643300*), and found that *OsPIN2* and *OsPIN10a* were especially expressed in the epidermal cells (Supplementary Figures 4A,B). Therefore, we concluded that ABA-promoted root hair elongation likely requires functional basipetal auxin transport.

### RNA-Seq Analyses of the ABA-Treated Root Tips

To obtain a global view of the differential expression of genes related to ABA-induced root hair elongation, the 5-day-old seedlings were treated without or with 0.5 μM ABA for 24 h, then the three different zones (SRH, LRH, and Tip) of root tips were collected to analyze transcript profiles (Supplementary Figure 3). We conducted two biological replicates and analyzed their repeatability by calculating the Pearson correlation coefficient (Supplementary Figure 5A), which indicates these results are consistent and repeatable.

To confirm the result that ABA promotes auxin transport and to validate the RNA-seq accuracy, we used RNA-seq to analyze the expression of *OsPINs, OsAUX*, and *PINOID* (*PID*). *Arabidopsis PID* encodes a serine/threonine protein kinase that regulates auxin redistribution through control of the subcellular localization of PINs (Robert and Offringa, 2008). As shown in Supplementary Figure 5B, ABA treatment promotes auxin transport by enhancing expression of *OsPINs, OsAUX*, and *OsPID*, especially in the Tip and LRH zones, consistent with the qRT-PCR (**Figure 4D**) and *OsPINs-GUS* staining results (**Figure 4E**). The results also showed a high correlation (*R*^2^ = 0.84, Supplementary Table 2) between the RNA-seq and qRT-PCR data.

### ABA Promotes Auxin Biosynthesis

**FIGURE 5:**
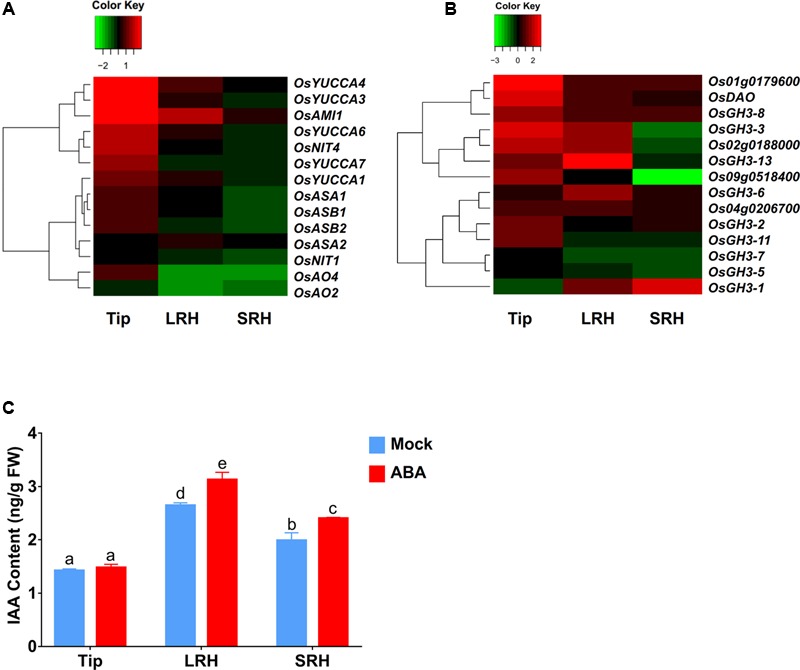
ABA promotes local auxin biosynthesis and accumulation in the LRH zone. **(A)** Auxin biosynthetic gene expression profile in Tip, LRH, and SRH under ABA treatments. **(B)** Auxin metabolic gene expression profile in Tip, LRH, and SRH under ABA treatments. **(C)** Quantification of IAA level in the Tip, LRH, and SRH under ABA treatments.

In addition to increasing auxin transport to the LRH zone, enhanced local auxin biosynthesis or reduced local auxin degradation could also increase local auxin concentrations, as indicated by the enhanced *DR5-GUS* expression in the LRH zone. Therefore, we first used our RNA-seq data to measure the expression levels of genes involved in auxin biosynthesis, including *OsYUCCAs* (Yamamoto et al., 2005), *OASA* (Kanno et al., 2004*), OASB, OsNIT, OsAO*, and *OsAMI*. These data showed that almost all of these auxin biosynthesis genes were upregulated after ABA treatment in the root tips (**Figure 5A** and Supplementary Table 4) and that *OsAMI1* (∼3.6-fold), *OsYUCCA4* (∼1.8-fold), and *OsYUCCA1* (∼1.4-fold) were upregulated in the LRH zone.

We then checked the expression of the genes encoding IAA-amido synthetases of the GH3 family, key enzymes involved in the conversion of active IAA to an inactive form via conjugation of IAA with amino acids, such as Asp, Ala, and Phe (Fu et al., 2011). We also examined the genes encoding members of the UDP glycosyltransferase (UGT) family and dioxygenase for auxin oxidation (Os*DAO*) (Bowles et al., 2006; Zhao et al., 2013; Kasahara, 2016). In rice, 10 of the 14 genes involved in IAA inactivation were upregulated in the tip zone, and 9 of them were also upregulated in the LRH but not expressed in the SRH (**Figure 5B** and Supplementary Table 4), suggesting that the enhanced auxin transport and biosynthesis in the Tip and LRH zones of rice roots are the major reasons for local auxin accumulation, and the enhanced auxin inactivation is most likely caused by negative feedback regulation.

We also measured the endogenous IAA levels in the Tip, LRH, and SRH zones of the rice root. As shown in **Figure 5C**, auxin accumulated in the LRH zone, but not in the Tip, and the IAA concentration in the LRH zone after ABA treatment was significantly higher than in the untreated roots. We should note that the *DR5-GUS* staining shows strong signal in the root tip, indicating high concentration auxin in the root tip. However, we measured the IAA concentration in Tip, LRH, and SRH, and found the different IAA concentration between “Tip” and the typical root tip as shown in Supplementary Figure 3. We would like clarify here that the defined “Tip” here includes not only the classical root tip region but also the meristematic zone, transition zone, and root hair zone under the LRH, which covers a larger region. In addition, we used the roots from the 5-day-old seedling, and the majority of auxin may be from the shoot by transport.

### A Number of Genes Regulated by Auxin Are Involved in Root Hair Elongation in Response to ABA

**FIGURE 6:**
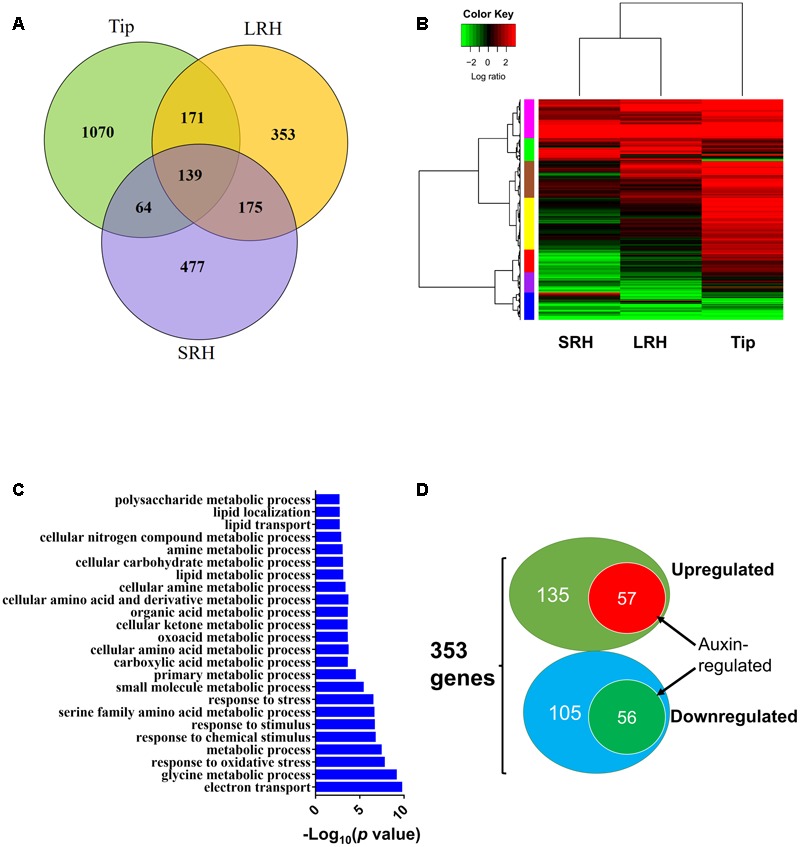
Differentially expressed genes after ABA treatment in the rice root tip (Tip), long root hair (LRH), and short root hair (SRH) zones. **(A)** Venn diagram showing the differentially expressed genes in the Tip, LRH and SRH zones in response to treatment with 0.5 μM ABA for 24 h. **(B)** Hierarchical clustering analysis of the differentially expressed genes in the Tip, LRH, and SRH zones with or without 0.5 μM ABA treatment for 24 hrs. **(C)** GO term analyses shows the biological processes (BPs) enriched in the LRH before and after ABA treatments. **(D)** Venn diagram showing the overlapping differentially expressed genes regulated by ABA and auxin in the LRH zone. The auxin-regulated genes were based on the RiceXPro database.

To extend our analysis, we globally analyzed the genes differentially regulated in response to exogenous ABA in the three regions of root tips. We identified 1444, 838, and 855 genes that were differentially expressed before and after ABA treatment in Tip, LRH, and SRH, respectively. Of these, 1070, 353, and 477 genes were altered specifically in Tip, LRH, and SRH, respectively (**Figure 6A**). Further clustering analysis of these differentially expressed genes in each zone indicated that most of the ABA upregulated genes are in the Tip, followed by the LRH, and the fewest in the SRH (**Figure 6B**). These results suggest that the rice root tip cells rapidly sense and respond to ABA.

To understand the overall biological processes that occurred in the LRH zone in response ABA treatment, we performed gene ontology (GO) enrichment analysis of the 353 differentially expressed genes in the LRH; this identified 24 significantly (FDR < 0.05) enriched GO terms (**Figure 6C**) in two big categories. The first category includes GO terms involved in stress responses, such as “response to oxidative stress,” “response to chemical stimulus,” “response to stimulus,” and “response to stress.” The second category includes GO terms involved in “metabolic process,” such as “glycine metabolic process,” “small molecule metabolic process,” “primary metabolic process,” “cellular amino acid and derivative metabolic process,” “lipid metabolic process,” “cellular carbohydrate metabolic process,” and “polysaccharide metabolic process.” Then, we found 19 stress-responsive genes specifically responding to ABA in LRH (Supplementary Table 3). Nine of the 19 stress-responsive genes encode peroxidases, suggesting that reactive oxygen species have important roles in root hair growth in response to ABA treatment.

Because of the importance of auxin in root hair elongation, to understand whether these differentially expressed genes are related to ABA-regulated root hair elongation, we checked whether auxin affects the 353 genes that are differentially expressed in the LRH by comparing the genes to the auxin-regulated genes in the RiceXpro database (Sato et al., 2013). As shown in **Figure 6D**, among the 192 ABA upregulated genes, 57 (∼29.7%) were also upregulated by auxin (**Table 1**), and 56 of the 161 ABA downregulated genes were also repressed by auxin (**Table 2**). This suggests that auxin plays an important role in ABA-promoted rice root hair elongation, and the 113 genes co-regulated by both ABA and auxin in the LRH zone may function directly in regulating root hair elongation. We found that *OsPIN9* and *OsPP2C 59* are upregulated, further supporting the importance of polar auxin transport in root hair elongation.

**Table 1:** ABA and Auxin upregulated genes specially changed in LRH under ABA treatment.

Gene_ID	Log_2_ (FC)	Gene description
OS01G0251400	inf	Os01g0251400 protein
OS11G0454200	inf	Dehydrin Rab16B
OS06G0279900	inf	Putative NBS-LRR class RGA
OS09G0109600	inf	Putative uncharacterized protein
OS12G0455000	7.06	Expressed protein
OS01G0802700	6.16	Probable auxin efflux carrier component 5
OS03G0432100	4.73	Similar to orthophosphate dikinase precursor
OS01G0113800	4.32	Putative rust resistance kinase Lr10
OS08G0101800	4.10	Putative uncharacterized protein B1147B12.20
OS04G0560100	3.98	OSJNBa0084K11.4 protein
OS03G0702100	3.55	Hypothetical protein
OS06G0698300	3.51	Probable protein phosphatase 2C 59
OS05G0424300	3.44	Putative cytochrome P450
OS05G0373900	3.38	putative peptide chain release factor subunit 1 (ERF1)
OS07G0142500	3.36	Early nodulin 75-like protein
OS07G0587300	3.13	Putative uncharacterized protein OJ1047_C01.8
OS07G0599300	3.12	Proline-rich protein family-like protein
OS07G0541200	3.05	Putative serine/threonine kinase protein
OS08G0412800	3.05	Os08g0412800 protein; putative uncharacterized protein OSJNBa0007M04.21
OS02G0466400	3.03	Similar to inositol phosphate kinase
OS07G0142700	2.95	*N*-hydroxycinnamoyl benzoyltransferase-like protein
OS03G0286900	2.93	Putative low-temperature induced protein
OS02G0631100	2.81	Putative uncharacterized protein B1250G12.8
OS06G0610800	2.81	Putative nucleoid DNA-binding protein cnd41, chloroplast
OS10G0345100	2.75	Multi antimicrobial extrusion protein MatE family protein
OS09G0517100	2.73	NB-ARC domain containing protein
OS04G0639100	2.70	Conserved hypothetical protein
OS07G0599600	2.63	Early nodulin 75-like protein
OS03G0807900	2.59	Expressed protein
OS07G0599900	2.54	Putative uncharacterized protein OJ1634_B10.117
OS01G0177900	2.52	ABC-2 type transporter domain containing protein
OS04G0301500	2.52	Helix-loop-helix DNA-binding domain containing protein
OS12G0154800	2.47	Germin-like protein 12-2
OS03G0820300	2.42	C2H2 transcription factor; putative Cys2/His2 zinc-finger protein; zinc finger protein ZFP182
OS07G0599500	2.38	Early nodulin 75-like protein
OS09G0468300	2.37	RING-H2 zinc finger protein ATL6-like
OS12G0154900	2.33	Putative germin-like protein 12-3
OS06G0164400	2.32	Basic helix-loop-helix dimerization region bHLH domain containing protein
OS09G0471200	2.30	EGF-like calcium-binding domain containing protein
OS01G0836600	2.28	Putative ATP-binding cassette transporter ABCG2
OS05G0573300	2.27	CTP synthase
OS08G0137800	2.26	Cupredoxin domain containing protein.
OS01G0692100	2.18	Uncharacterized protein
OS07G0676600	2.17	Putative uncharacterized protein OJ1167_G06.116; cDNA, clone
OS07G0599700	2.14	Proline-rich protein family-like protein
OS01G0952500	2.12	Type A response regulator 4
OS07G0584100	2.07	Probable serine/threonine-protein kinase WNK5
OS03G0762100	2.06	Expressed protein
OS05G0195200	2.06	Zinc finger CCCH domain-containing protein 35
OS11G0521000	2.05	GDSL-like lipase/acylhydrolase family protein
OS03G0326200	2.05	Phospholipid-transporting ATPase 1
OS07G0271000	2.03	Putative GDP dissociation inhibitor
OS10G0392400	1.95	ZIM motif family protein
OS06G0274800	1.86	Class III peroxidase 77; putative peroxidase 49
OS12G0154700	1.85	Germin-like protein 12-1
OS03G0281500	1.85	Protein kinase
OS02G0686700	1.78	Putative uncharacterized protein OJ1717_A09.39

*Shown are the log_2_ fold change values (log_2_ FC) for genes commonly induced (>1.5) by ABA with adjusted *p*-values < 0.05.*

**Table 2:** ABA and Auxin downregulated genes specially changed in LRH under ABA treatment.

Gene_ID	Log_2_ (FC)	Gene description
OS01G0329900	-1.55	Putative early nodule-specific protein ENOD8
OS01G0668100	-1.57	Arabinogalactan protein-like
OS02G0640300	-1.62	Steroid membrane binding protein-like
OS10G0122600	-1.63	Similar to predicted protein
OS01G0195400	-1.63	Putative uncharacterized protein P0001B06.31
OS12G0443500	-1.65	UDP-glucose 6-dehydrogenase 4
OS09G0353700	-1.71	Similar to Leucoanthocyanidin dioxygenase
OS09G0274900	-1.72	Putative uncharacterized protein OJ1031_C12.40
OS01G0805900	-1.73	Tubulin beta-4 chain
OS02G0187800	-1.77	Cinnamyl alcohol dehydrogenase 2
OS04G0491500	-1.77	TGF-beta receptor, type I/II extracellular region family protein
OS06G0680500	-1.79	Glutamate receptor
OS12G0225900	-1.89	NADP-dependent oxidoreductase P1
OS11G0270000	-1.89	Crotonase, core domain containing protein
OS04G0438200	-1.98	Similar to H0315A08.10 protein
OS01G0770700	-1.99	Copper transporter 1
OS11G0138900	-2.02	Alpha/beta hydrolase fold-3 domain containing protein
OS02G0684100	-2.04	Putative steroid sulfotransferase
OS03G0760500	-2.05	Cytochrome P450 family protein
OS03G0317300	-2.06	Eukaryotic aspartyl protease family protein
OS06G0592400	-2.08	Similar to cytosolic aldehyde dehydrogenase RF2C
OS02G0753000	-2.12	Probable trehalose-phosphate phosphatase 4
OS05G0427900	-2.12	Similar to DnaJ-like protein
OS10G0130800	-2.20	Conserved hypothetical protein
OS12G0260500	-2.21	Similar to oxidoreductase, short chain dehydrogenase/reductase family protein, expressed
OS03G0831400	-2.22	Expressed protein
OS11G0290600	-2.24	Conserved hypothetical protein
OS10G0552300	-2.35	Conserved hypothetical protein
OS04G0472200	-2.36	Similar to H0418A01.11 protein
OS04G0659200	-2.37	Hypothetical protein
OS01G0916100	-2.50	Similar to loricrin
OS10G0320100	-2.50	Flavonoid 3′-hydroxylase; flavonoid 3′-monooxygenase
OS10G0546100	-2.54	Pollen proteins Ole e I family protein
OS02G0574000	-2.56	Similar to monosaccharide transporter 1
OS03G0699700	-2.58	Linoleate 9S-lipoxygenase 1
OS11G0708100	-2.64	Similar to laccase-22
OS01G0263000	-2.65	Peroxidase superfamily protein
OS02G0767300	-2.76	Putative flavonol synthase
OS05G0134800	-2.82	Class III peroxidase 67
OS03G0143900	-2.82	Disease resistance-responsive family protein
OS02G0280200	-2.92	Similar to xyloglucan endotransglucosylase/hydrolase protein 26
OS01G0899700	-2.93	Putative extensin
OS06G0335900	-2.93	Putative Xet3 protein
OS07G0499500	-3.00	Putative peroxidase prx15
OS05G0372900	-3.06	Putative uncharacterized protein
OS03G0368000	-3.13	Class III peroxidase 42; peroxidase family protein
OS08G0503000	-3.23	Hypothetical protein
OS07G0542900	-3.29	Putative phytocyanin
OS04G0674800	-3.41	Endoglucanase 13
OS05G0382900	-3.47	Annexin
OS03G0608000	-3.75	Expressed protein
OS12G0163700	-4.10	Similar to Actin 7
OS01G0370000	-4.51	Putative 12-oxophytodienoate reductase 9
OS01G0550800	-4.69	Putative ZmEBE-1 protein
OS10G0109300	-5.35	Class III peroxidase 125
OS01G0216000	-8.26	Putative esterase

*Shown are the log_2_ fold change values (log_2_ FC) for genes commonly repressed (< -1.5) by ABA with adjusted *p*-values < 0.05.*

To identify root hair-specific genes, we also screened the promoter regions of the 113 genes co-regulated by ABA and auxin (2000 bp upstream of the start codon) for the RHE sequence “WHHDTGNNN(N)KCACGWH” (where W = A/T, H = A/T/C, D = G/T/A, K = G/T, and N = A/T/C/G), as previously described (Won et al., 2009). We found 69 RHEs in 51 genes, with 13 genes carrying two or more RHEs (**Table 3** and Supplementary Table 5). Because we lack root hair-specific gene expression data, we predicted whether these genes are specifically expressed in epidermal cells by searching the rice microarray expression database (RiceXPro) (Supplementary Table 5). These results indicated that 35 out of the 51 genes containing the RHE are highly expressed in epidermal cells in rice root tips.

**Table 3:** Distribution of RHE motif in the upregulated genes promoter between mock and ABA treatment in LRH.

								Epidermal expression
Sequence name	Strand	Start	End	*p*-value	Matched sequence	Log_2_ (FC)	Description	in root tip
Os11g0454200	+	1724	1739	7.25E-05	TACGTGGCAGCAGGTT	inf	Dehydrin RAB 16B.	Yes
Os12g0455000	-	1560	1576	8.61E-05	TCCTTGCATTGCAAGTC	7.06262	Conserved hypothetical protein.	Yes
Os04g0560100	+	1050	1065	5.94E-05	ATTTTGTCTACACGAA	3.98115	Cytochrome P450 family protein.	Yes
Os07g0599300	-	173	188	1.67E-05	TTTTTGCCAGCACGAG	3.12285	Conserved hypothetical protein.	Yes
Os08g0412800	+	1181	1196	5.90E-05	ACTTTGTATACACGTA	3.04954	Protein of unknown function DUF1262 family protein.	Yes
Os10g0345100	+	1618	1634	3.40E-05	TCCACGCGTCGCACGAC	2.75364	Multi antimicrobial extrusion protein MatE family protein.	Yes
Os07g0599600	+	1634	1650	2.89E-05	TCTTTGTCATGCACGGC	2.63352	Early nodulin 75-like protein	Yes
Os07g0599600	-	753	769	8.05E-05	ACAATGCGTGGGACGAA	2.63352	Early nodulin 75-like protein	Yes
Os03g0820300	-	882	897	5.41E-05	TCCACGCACGCACGTA	2.41894	Similar to ZPT2-14. C2H2 transcript factor.	Yes
Os03g0820300	+	1926	1942	2.74E-05	TCCTTGGCAAACACGTA	2.41894	Similar to ZPT2-14. C2H2 transcript factor.	Yes
Os07g0599500	+	1626	1642	3.99E-06	TCCTTGTCATGCACGAT	2.38247	Conserved hypothetical protein.	Yes
Os12g0154900	+	387	403	7.35E-05	CAATTGTGTTTCACGTA	2.32817	Similar to Germin-like protein precursor.	Yes
Os08g0137800	-	1227	1243	4.70E-06	TCCTTGCCATTCACGAA	2.25575	Cupredoxin domain containing protein.	Yes
Os08g0137800	-	401	417	5.08E-05	ATGTTGTTTTTCACGAT	2.25575	Cupredoxin domain containing protein.	Yes
Os07g0599700	+	357	373	6.37E-05	TCCGCGTACAGCACGTA	2.1417	Similar to Surface protein PspC.	Yes
Os07g0584100	+	1043	1059	6.37E-05	AACGCGGACTGCACGTT	2.06878	Similar to MAP kinase-like protein.	Yes
Os11g0521000	-	672	688	7.18E-05	TCCTTGCCACGCATGTT	2.05367	Lipolytic enzyme, G-D-S-L family protein.	Yes
Os07g0271000	+	67	83	4.69E-05	AAAGTGGGACGCTCGTC	2.03135	Similar to GDP dissociation inhibitor protein OsGDI1.	Yes
Os10g0392400	+	1507	1523	6.60E-05	ACAGTGTGAGGCATGAC	1.95131	ZIM domain containing protein.	Yes
Os10g0392400	+	379	395	7.26E-05	CATATGAAATGCACGTT	1.95131	ZIM domain containing protein.	Yes

## Discussion

This study provides several lines of evidence to demonstrate that in rice, ABA promotes root hair elongation by regulating auxin transport and biosynthesis in specific zones of the roots. First, exogenous ABA treatment enhances root hair elongation in rice root tip and inhibit ABA biosynthesis also repress the root hair elongation. It has been reported that low concentration (0.1 μM) of exogenous ABA can promote root elongation, but high concentrations (>0.5 μM) of ABA inhibits root elongation (Ghassemian et al., 2000; Rowe et al., 2016). We also found that 0.1 μM ABA inhibit root hair elongation, while 0.5 μM and 2 μM ABA enhanced rice root hair elongation. One explanation is that feedback regulation is a common regulatory mechanism for phytohormonal signaling, so different concentrations of ABA may reflect the different levels and results of feedback. Second, examination of transgenic lines overexpressing *OsABIL2* or *SAPK10*, key components in rice ABA signaling showed that ABA-promoted root hair elongation is dependent on the major PYR/PP2C/SnRK signaling pathway. The ABA-insensitive *OsABIL2-OE* line showed decreased root hair elongation, and the ABA-hypersensitive line *SAPK10-OE* showed enhanced root hair elongation. Third, our analysis demonstrated that auxin acts downstream of ABA signaling to promote root hair elongation, which depends on the ABA-regulated polar auxin transport and local auxin biosynthesis. Our results consistent with previous studies that ABA and auxin functionally interact in roots (Rock and Sun, 2005; Xu et al., 2013). For example, ABA accumulation promotes auxin transport in root tips and enhance proton secretion for maintaining root growth under moderate water stress (Xu et al., 2013). Fourth, RNA-seq analysis and RHE screening identified 35 genes that respond strongly to ABA and auxin and may also be specifically expressed in root hair cells in the rice LRH zone. These genes may have important functions in regulating root hair elongation in rice. The last few years have been seen significant progress being made in uncovering the mechanisms that are involved in root hair development in rice, but major knowledge gaps still persist, especially compared with *Arabidopsis*, in which 138 genes related to root hair development have already been identified. While 8 genes that are involved in root hair development^4^ (Marzec et al., 2015). Our results provide 35 RHE-contained genes as candidate genes to regulate rice root hair development.

Polar auxin transport apparently plays a critical role in the ABA signaling-regulated root hair elongation in rice. Treatment with the auxin transport inhibitor NPA and staining of *DR5-GUS* lines demonstrated that ABA-promoted root hair elongation depends on polar auxin transport. ABA signaling is required for the ABA-regulated redistribution of auxin. In addition, auxin homeostasis in the root hair cell is critical for root hair elongation. In *Arabidopsis*, root hair-specific expression of auxin efflux carriers such as *PINs* (*PIN1-4, PIN7*, and *PIN8*) strongly suppresses root hair length, suggesting that auxin efflux carriers inhibit root hair elongation by depleting auxin in the root hair cell (Lee and Cho, 2006, 2013; Cho et al., 2007). Redistribution of auxin from its concentration maximum to epidermal cells requires the activity of PIN2, AUX1 and other carriers (Swarup et al., 2005; Ikeda et al., 2009). We showed that ABA not only significantly induces *OsPINs* and *OsAUX1* mRNA levels, as confirmed by qRT-PCR and RNA-seq analysis, but also strongly induces the ectopic expression of *OsPIN2* and *OsPIN10a* in the LRH zone. Because *OsPIN2* and *OsPIN10a* are specifically expressed in the epidermal cells, indicating that ABA promotion of root hair elongation requires functional basipetal auxin transport. Consistent with our results, other workers have reported that under osmotic stress in an ABA-regulated manner enhanced *PIN2* expression level in *Arabidopsis* root (Rowe et al., 2016).

Besides polar auxin transport, local auxin biosynthesis was also shown to modulate gradient-directed planar polarity in root hair development in *Arabidopsis* (Ikeda et al., 2009). We found that the ABA-enhanced accumulation of auxin in the LRH is also a consequence of the upregulation of local auxin biosynthesis in the Tip and LRH zones. First, ABA upregulates expression of almost all auxin biosynthetic genes in the Tip zone and upregulates *OsAMI1, OsYUCCA4*, and *OsYUCCA1* in the LRH zone. Second, examination of negative feedback mechanisms showed that 10 of the 14 genes involved in the inactivation of IAA were upregulated in the Tip zone and 9 were also upregulated in the LRH zone, indicating that auxin biosynthesis was enhanced in the Tip and LRH zones. Third, direct measurement of the endogenous IAA levels in the Tip, LRH, and SRH zones of the rice roots directly demonstrated the ABA-enhanced auxin accumulation in rice roots. However, the IAA concentration in the Tip region was identical before and after ABA treatment. A reasonable explanation is that ABA-promoted basipetal auxin transport may lead to quick auxin flow into the LRH region. Alternatively, the drastic increase of the expression of genes encoding IAA inactivation enzymes in the Tip region promoted a rapid conversion of IAA to inactive forms.

**FIGURE 7:**
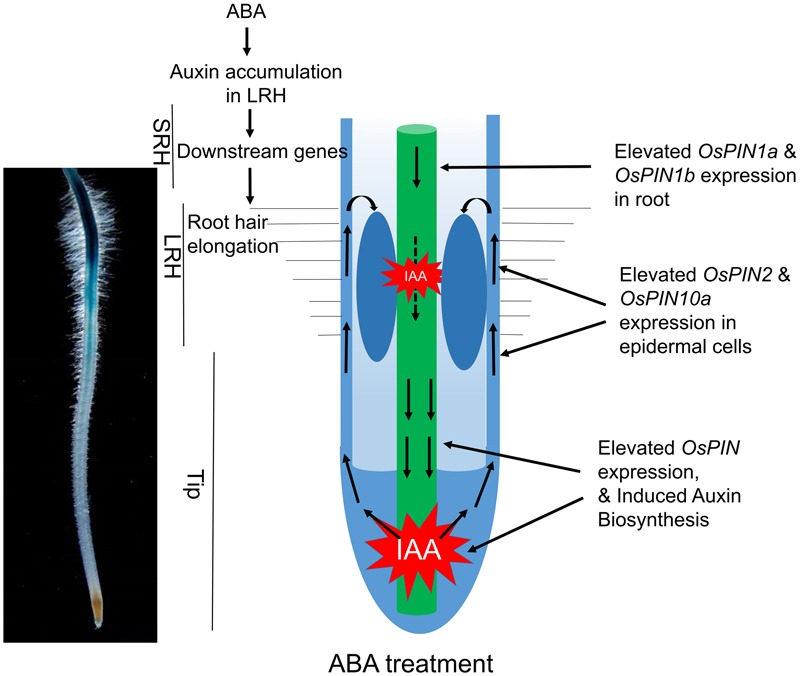
A proposed model of how ABA promotes auxin biosynthesis and transport to regulate root hair elongation. ABA signaling promotes local auxin biosynthesis in the Tip and LRH regions, and regulates auxin basipetal transport, which result in auxin accumulation in the LRH region. The high concentration of auxin regulates downstream gene expression to promote root hair elongation.

Our current data and previous discoveries suggested a model for the root hair elongation regulated by ABA in rice (**Figure 7**). ABA signaling promotes auxin biosynthesis in the Tip and LRH regions, and further enhanced the redistribution auxin through auxin basipetal transport to accumulate auxin in the LRH region. The high concentration of IAA activates downstream gene expression to promote root hair elongation. In the future, we plan to investigate the specific components in the ABA signaling pathway that directly regulate expression of genes involved in auxin transport and biosynthesis. In addition, the zone-specific genes identified in this study provide a great opportunity and resource to further understand how ABA regulates root hair elongation. In addition, the root hair region seems moving down during ABA treatment. We predicted this maybe caused by local auxin accumulation. According to our results, ABA promotes auxin acropetal and basipetal transport in rice tip and results in auxin local accumulation. As the applied ABA concentration increases, auxin may accumulate in the region much close to rice root tip, which lead to the move down of long root hair region. In addition, the inhibited root elongation by high concentration ABA may also result in the long root hair region closer to the root tip.

## Author Contributions

TW and XW conceived the research and planned the experiments; TW performed all of the experiments; CL provided SAPK10 and OsABIL2 transgenic plants; ZW performed the RNA-seq data analysis; YJ and HW helped to prepare material for RNA-seq; CM provided OsPIN-GUS transgenic plants; SS put forward improvement advise. TW and XW wrote the manuscript.

## Conflict of Interest Statement

The authors declare that the research was conducted in the absence of any commercial or financial relationships that could be construed as a potential conflict of interest.

## Footnotes

1 https://imagej.nih.gov/ij/index.html

2 http://www.ncbi.nlm.nih.gov/tools/primer-blast/

3 http://ricexpro.dna.affrc.go.jp

4 www.iroothair.org

## Abbreviations

ABA: abscisic acid
IAA: indole-3-acetic acid
NAA: naphthaleneacetic acid
NPA: 1-*N*-naphthylphthalamic acid
RAM: root apical meristem
RHE: root hair regulatory element
SEM: scanning electron microscopy
