# Supplementary material for: Abscisic Acid Regulates Auxin Homeostasis in Rice Root Tips to Promote Root Hair Elongation
Source: Front Plant Sci. 2017 Jun 28;8:1121. doi: 10.3389/fpls.2017.01121 (PMC5487450; doi:10.3389/fpls.2017.01121)
Supplement: Supplementary file 6 [file Data_Sheet_1.docx]

Supplementary Material

**Abscisic acid regulates auxin biosynthesis and transport in rice root tips to promote root hair elongation**

**Tao Wang^1, 2^, Chengxiang Li^1^, Zhihua Wu^2^, Yancui Jia^2^, Hong Wang^2^, Shiyong Sun^2^, Chuanzao Mao^3^, and Xuelu Wang^2*^**

*** Correspondence:** Xuelu Wang, [xlwang@mail.hzau.edu.cn](mailto:xlwang@mail.hzau.edu.cn)

# Supplementary Figures and Tables

## Supplementary Figures

**
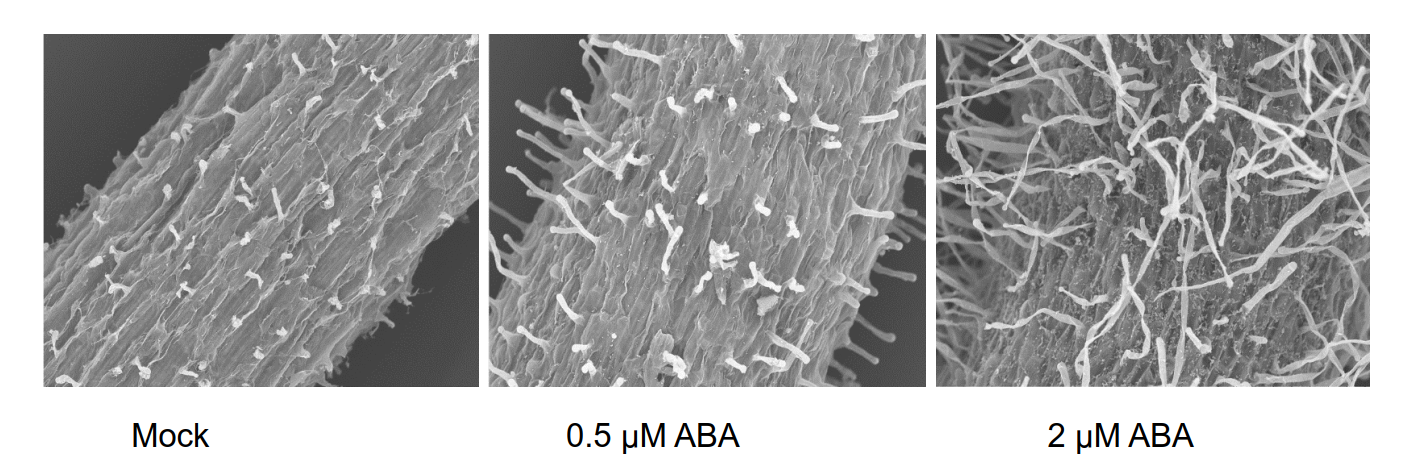
****Supplementary Figure 1.** **Root hair length.**  Images show scanning electron micrographs of the region 3 mm from the root apex.

**
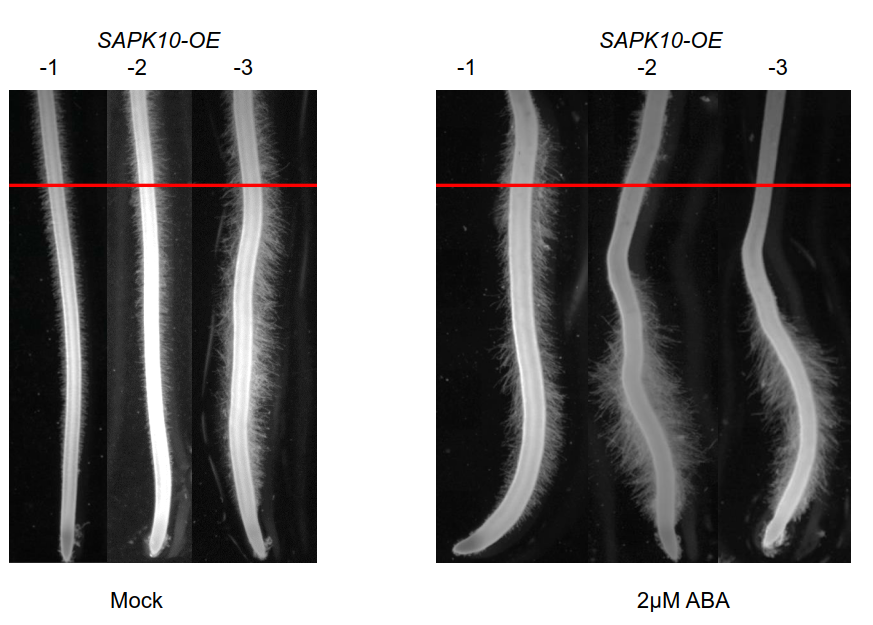
**

**Supplementary Figure 2. Root hair morphology of *SAPK10-OE* transgenic lines.** Red line is 4 mm from the root apex.


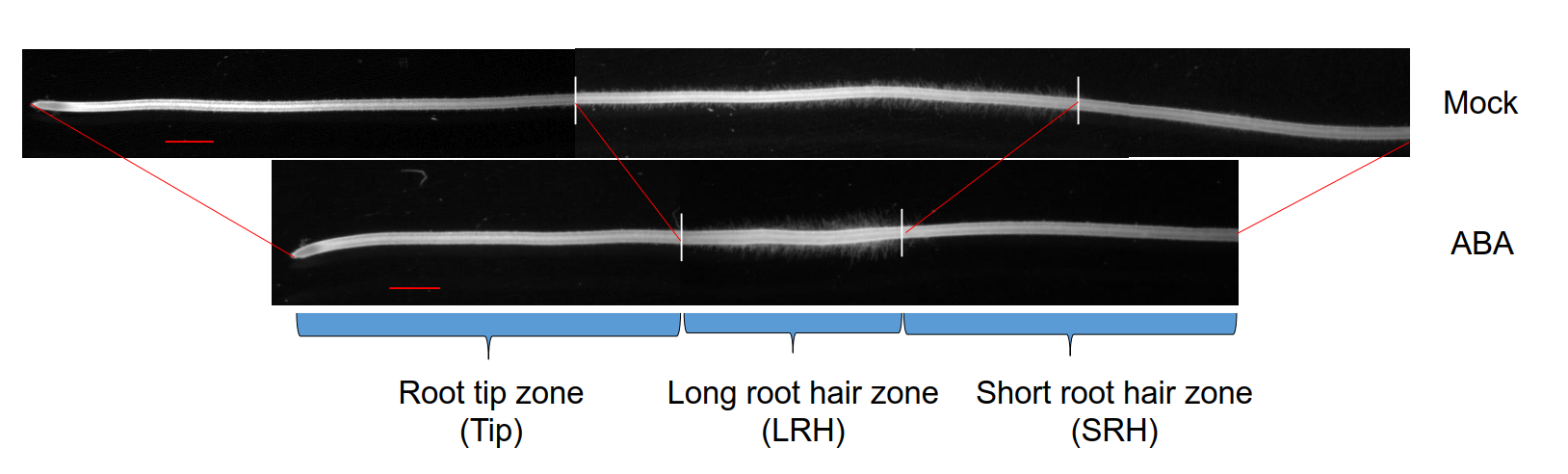


**Supplementary Figure 3.** **Zones of the root tips.** Root tips are divided into 3 zones according to the root hair length: Root tip zone (Tip), Long root hair zone (LRH), and Short root hair zone (SRH).


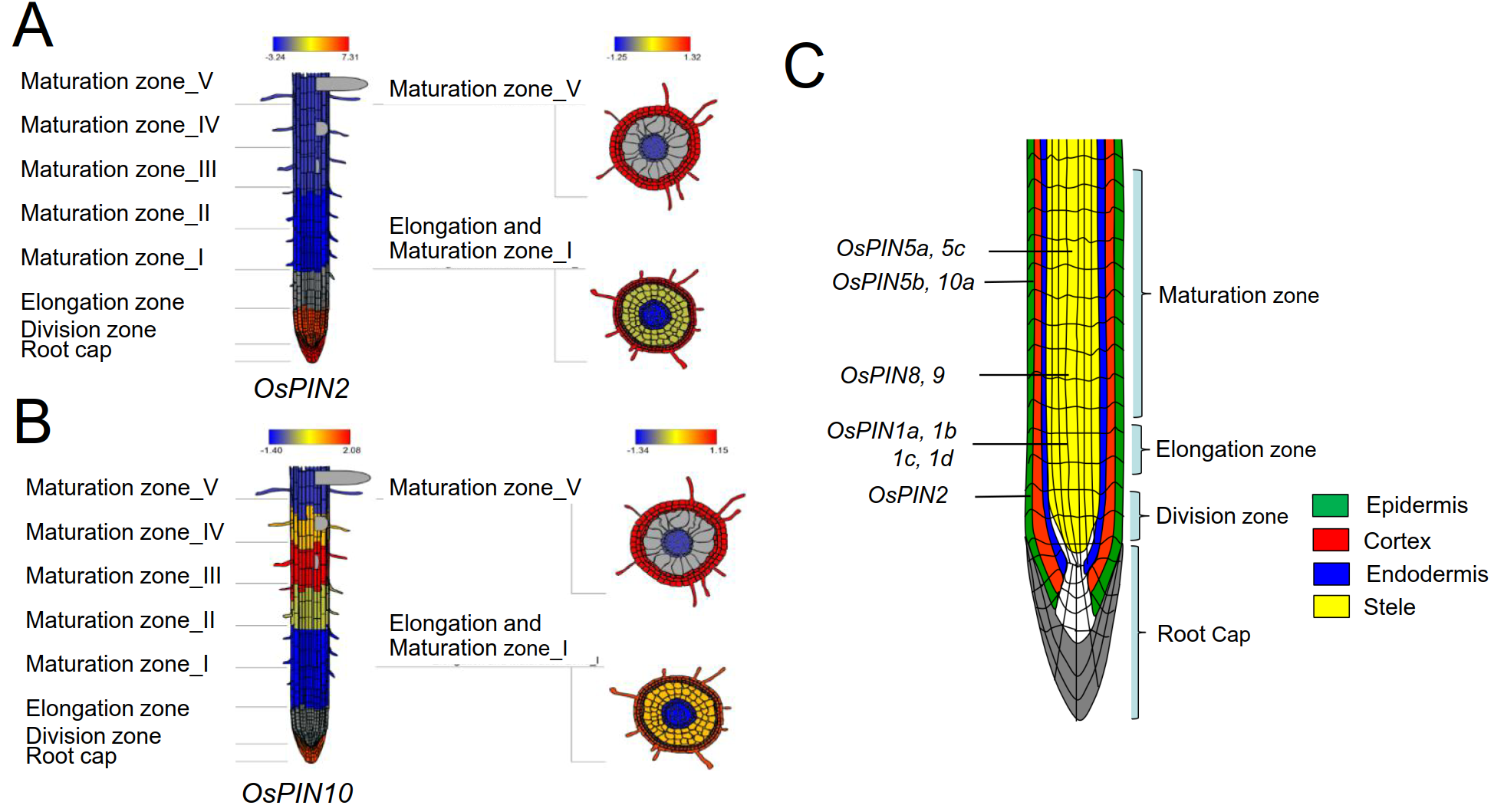


**Supplementary Figure 4.** ***OsPIN2* and *OsPIN10a* are specifically expressed in the epidermal cells.** The localization of the expression domains of *OsPINs* in roots, according to the RiceXPro database.


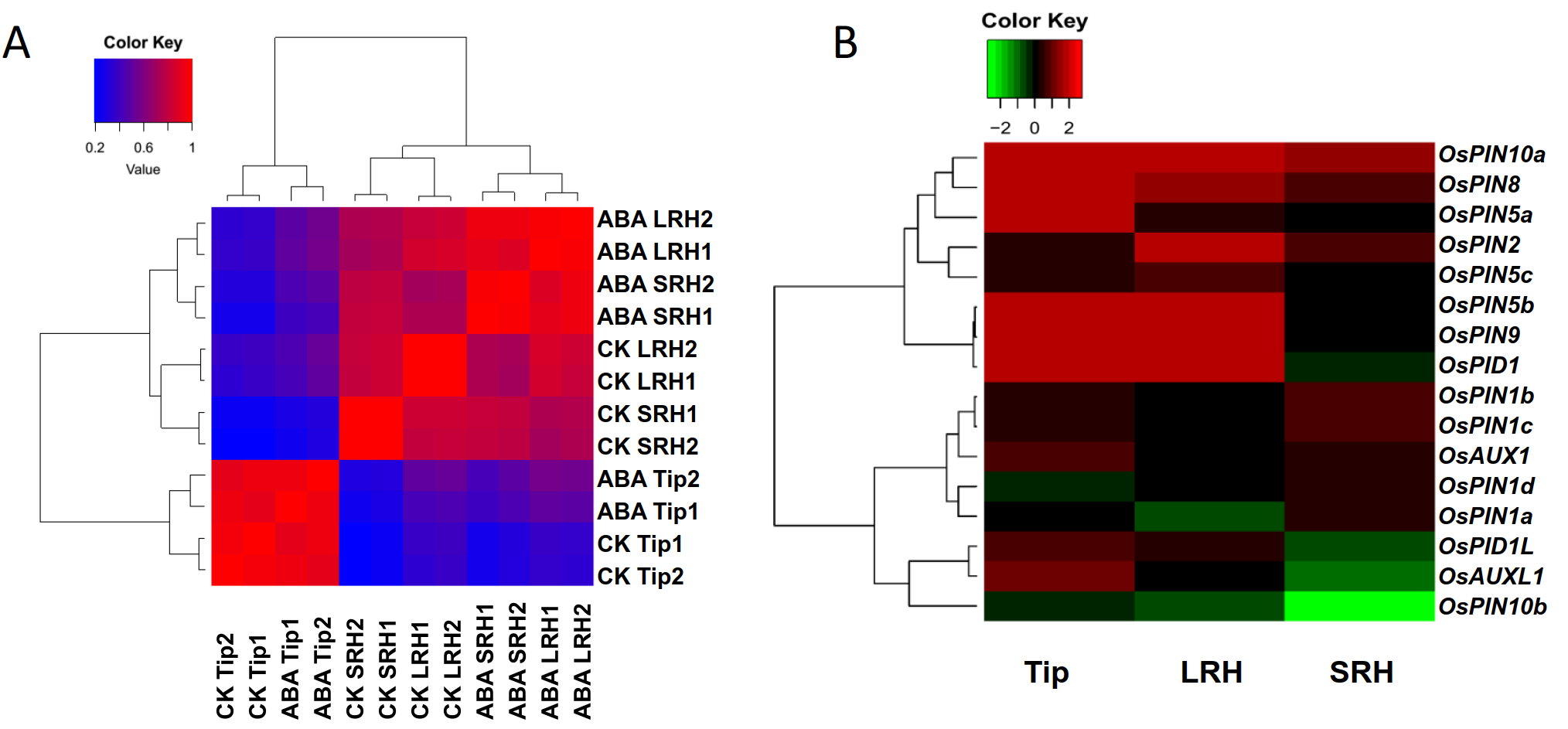


**Supplementary Figure 5.** **ABA promotes the expression of genes related to auxin transport in the root tips.**

## Supplementary Tables

**Supplementary Table 1. List of primers used in this study.**

**Supplementary Table 2. The expression data for *OsPINs.***

**Supplementary Table 3. Differentially expressed genes responding to stresses in the LRH region.**

**Supplementary Table 4. Auxin biosynthetic gene and metabolic gene changes under ABA treatment by RNA-seq analysis.** Table Showed the log_2_ fold change values (log_2_ FC) for genes.

**Supplementary Table 5. Distribution of the RHE motif in the promoter regions of the genes that are differentially expressed in the LRH after ABA treatment**
